# Gene expression information improves reliability of receptor status in breast cancer patients

**DOI:** 10.18632/oncotarget.20474

**Published:** 2017-08-24

**Authors:** Michael Kenn, Karin Schlangen, Dan Cacsire Castillo-Tong, Christian F. Singer, Michael Cibena, Heinz Koelbl, Wolfgang Schreiner

**Affiliations:** ^1^ Section of Biosimulation and Bioinformatics, Center for Medical Statistics, Informatics and Intelligent Systems (CeMSIIS), Medical University of Vienna, A-1090 Vienna, Austria; ^2^ Translational Gynecology Group, Department of Obstetrics and Gynecology, Comprehensive Cancer Center, Medical University of Vienna, A-1090 Vienna, Austria; ^3^ Department of General Gynecology and Gynecologic Oncology, Medical University of Vienna, A-1090 Vienna, Austria

**Keywords:** gene expression, breast cancer, receptor status, data science, mathematical oncology

## Abstract

Immunohistochemical (IHC) determination of receptor status in breast cancer patients is frequently inaccurate. Since it directs the choice of systemic therapy, it is essential to increase its reliability.

We increase the validity of IHC receptor expression by additionally considering gene expression (GE) measurements. Crisp therapeutic decisions are based on IHC estimates, even if they are borderline reliable. We further improve decision quality by a responsibility function, defining a critical domain for gene expression. Refined normalization is devised to file any newly diagnosed patient into existing data bases. Our approach renders receptor estimates more reliable by identifying patients with questionable receptor status. The approach is also more efficient since the rate of conclusive samples is increased. We have curated and evaluated gene expression data, together with clinical information, from 2880 breast cancer patients. Combining IHC with gene expression information yields a method more reliable and also more efficient as compared to common practice up to now.

Several types of possibly suboptimal treatment allocations, based on IHC receptor status alone, are enumerated. A ‘therapy allocation check’ identifies patients possibly miss-classified. Estrogen: false negative 8%, false positive 6%. Progesterone: false negative 14%, false positive 11%. HER2: false negative 2%, false positive 50%. Possible implications are discussed.

We propose an ‘expression look-up-plot’, allowing for a significant potential to improve the quality of precision medicine.

Methods are developed and exemplified here for breast cancer patients, but they may readily be transferred to diagnostic data relevant for therapeutic decisions in other fields of oncology.

## INTRODUCTION

The selection of an optimum breast cancer therapy has to include the expression of estrogen receptors (ER), progesterone (PGR) and human epidermal growth factor 2 (HER2) receptor proteins in an individual patient. The reliable assessment of these 3 receptor status is hence mandatory for optimum individualized therapy.

Although immunohistochemistry (IHC) is considered the gold standard for status determination, doubts have been raised [1–3] by reported differences between readers repeatedly examining the very same data. While almost complete concordance was found for ER^-^-status, up to 20% of patients assigned ER^+^- status may be erroneously classified, according to reports in the literature [4, 5]. Of note, we also found a considerable number of ER^-^ that might be misclassified.

First, correct assignment of ER-status is most important for the individualized choice of treatment [6]. As gene expression measurements can be achieved by different techniques, we investigate if the assessment of receptor status could be improved by additionally exploiting gene expression data.

Second, gene expression signatures for the prediction of individual therapeutic outcome have been established [7–11] and biomarker discovery methods developed [12–14], as recently reviewed [4, 15]. Each of these signature-algorithms includes receptor status as decisive variables upon which calculated prognosis crucially depends. Correct prognostic algorithms can thus be developed only on the basis of reliable receptor status [16].

Hence, it is of paramount value for both, patient treatment and research, to increase the reliability or even impute receptor status by the use of additional information, e.g., gene expression measurements [17–20]. In the present work we restrict ourselves to a single measurement platform for gene expression (Affymetrix U133A+2.0), in order to avoid inter-platform batch effects.

The present work deals with three receptors, ER, PGR and HER2.

## RESULTS

For the present work we used and curated the datasets listed in Table 1.

**Table 1:** Expression datasets used

GEO Accession number	Citation	Number of samples						
		N_sample_	ER-	ER+	PGR-	PGR+	HER2-	HER2+
GSE5460	[36]	29	11	18	0	0	21	8
GSE11001	[37]	30	12	18	16	14	23	7
GSE12777	[38]	51	0	0	0	0	34	17
GSE16179	[39]	18	0	0	0	0	0	18
GSE16391	[40]	55	0	55	0	0	42	3
GSE16446	[41]	120	0	0	0	0	90	28
GSE18728	[42]	61	29	32	36	25	44	17
GSE18864	[43]	84	53	31	53	31	64	18
GSE19615	[44]	115	45	70	51	64	79	36
GSE20685	[45]	327	0	0	0	0	0	0
GSE20711	[46]	88	45	42	0	0	62	26
GSE23177	[47]	116	0	116	0	0	116	0
GSE26639	[48]	226	88	138	128	95	145	81
GSE27120	[49]	28	4	24	12	16	26	2
GSE29431	[50]	54	0	0	0	0	12	41
GSE31448	[51]	353	162	188	154	167	234	31
GSE32646	[52]	115	44	71	70	45	0	0
GSE42568	[53]	104	34	67	0	0	0	0
GSE43365	[54]	111	18	93	34	77	96	13
GSE48390	[55]	81	28	53	0	0	47	34
GSE50948	[56]	156	104	52	121	35	42	114
GSE58812	[57]	107	107	0	107	0	107	0
GSE61304	[58, 59]	58	25	28	23	22	0	0
GSE71258	[60]	128	48	51	61	38	77	22
GSE76124	[61] [62]	198	198	0	198	0	198	0
GSE76274	[61] [62]	67	18	49	25	42	28	22
**Total**		**2880**	**1073**	**1196**	**1089**	**671**	**1587**	**538**

GSE-numbers refer to GEO [25, 26], using Affymetrix platform U133A+2.0. Number of samples are given for each study: in total (N_sample_) and also separately for receptor positive and receptor negative patients according to IHC measurements.

### Receptor status obtained from gene expression by conventional methods

**Figure 1:**
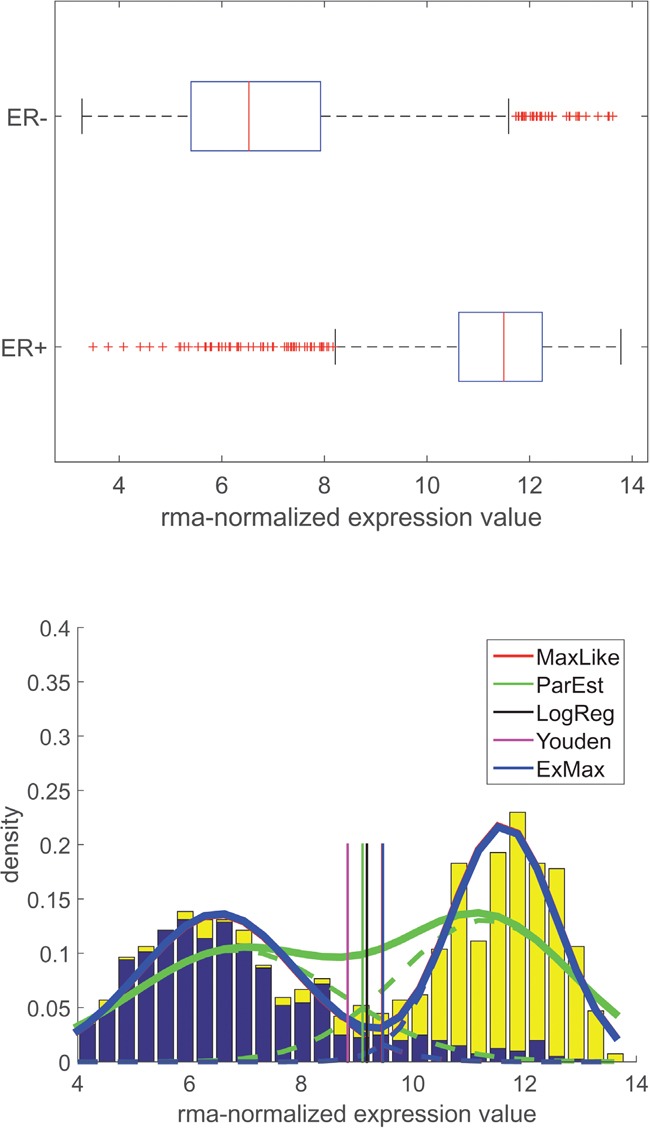
Distribution of gene expression for estrogen-receptor (ESR1): Data, models and thresholds for Affymetrix platform U133A+2.0 X-axis: gene expression. Upper panel: Boxplots for expression values of receptor gene *ESR1* (Affymetrix-probe set 205225_at), grouped according to IHC-estimate ($\text{ER}+≜\;{\text{ER}}_{\text{IHC}}^{+}$, $\text{ER}-≜\;{\text{ER}}_{\text{IHC}}^{-}$). Lower panel: Y-axis: probability density. Normalized histograms for expression values (blue: ${\text{ER}}_{\text{IHC}}^{-}$, yellow: ${\text{ER}}_{\text{IHC}}^{+}$). Estimated probability density functions (PDFs) modelled as sums of two normal distributions. PDF obtained ‘classically’ from means and standard deviations: green curves. Dashed lines: individual distributions for ${\text{ER}}_{\text{IHC}}^{+}$ and ${\text{ER}}_{\text{IHC}}^{-}$. Solid: superposition. For methods used see legend. Green dashed curves represent portions for ${\text{ER}}_{\text{IHC}}^{+}$ and ${\text{ER}}_{\text{IHC}}^{-}$ - patients (integrals < 1), adding up to the solid green curve (integral = 1). Cut-points (vertical lines) resulting from different computational approaches (see legend and text), discriminating receptor negative (left) and receptor positive (right) patients. Note that cut-points for MaxLike and ExMax coincide (only ExMax is shown in the figure).

Given the IHC-estimate of a receptor-status, e.g. $ER_{\text{IHC}}^{-}$ or $ER_{\text{IHC}}^{+}$, two distributions of expression values ($x_{i}$, *i* = 1,…, *N*_sample_, number of patients/samples) of the corresponding gene (e.g. ESR1. see Table 2) were estimated, see Figure 1. These plots show results for the estrogen receptor and also illustrate the computational procedure, see section ‘Methods and models for expression of receptor genes’.

**Table 2:** Probe sets for receptor genes ER, PGR and HER2 for the Affymetrix platform U133A+2.0

Receptor	ER	PGR	HER2
Probeset ID	205225_at	208305_at	216836_s_at
HUGO Nomenclature	ESR1	PGR	ERBB2
ENTREZID	2099	5241	2064

### Obtaining receptor status ROC curves

**Figure 2:**
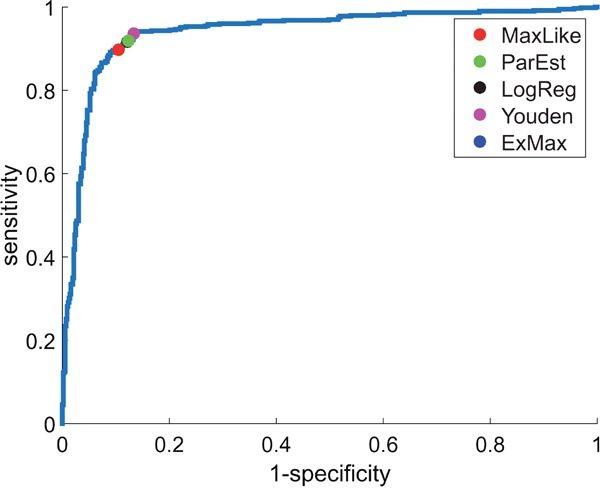
ROC-curve for estrogen-receptor gene (*ESR1*) . ROC-curve from IHC-estimates (e.g.: $ER_{\text{IHC}}^{+}$, $ER_{\text{IHC}}^{-}$) and RMA-normalized expression values of gene *ESR1* (Affymetrix-probe set 205225_at) yields AUC = 0.94 for U133A+2.0. Several concepts have been applied (see legend) to obtain thresholds for discrimination between ER^+^ and ER^-^. Sensitivity and specificity of each threshold can be read off the diagram axes.

As a prerequisite, we assumed IHC-estimates (e.g.: $ER_{\text{IHC}}^{+}$, $ER_{\text{IHC}}^{-}$) to be ‘true’ and computed a ROC-curve (blue curve in Figure 2) by classifying a patient as receptor positive/negative if the expression value, $x_{i}$, of gene *ESR1* was above/below a running threshold, $\hat{x}$. The area under the curve resulted as AUC = 0.94.

### Cut-points resulting from models of receptor gene expression

**Figure 3:**
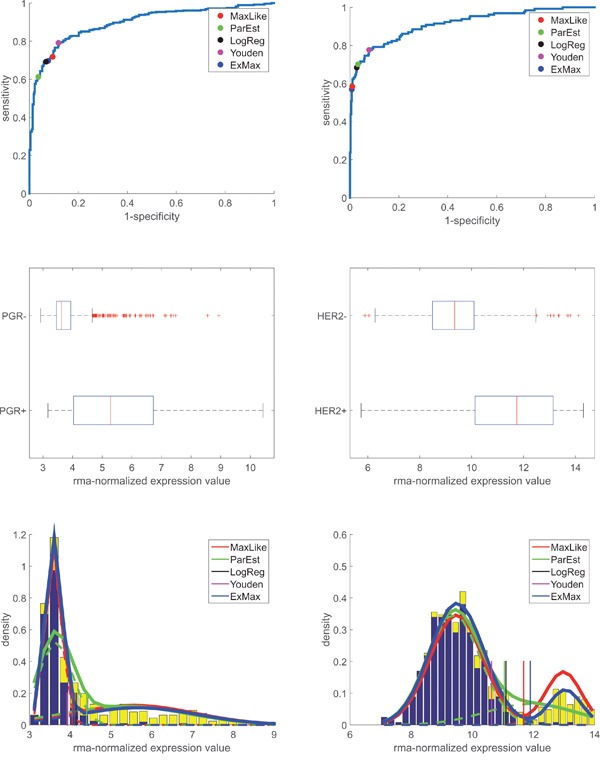
ROC-curves and probability density (distribution) of PGR and HER2-receptor genes’ expression (*PGR* and *ERBB2*, respectively): Data, models and thresholds Left panels: PGR. Right panels: HER2. Upper panels: ROC-curves constructed from PGR- and HER2-values from IHC and RMA-normalized expression values of genes PGR and *ERBB2* (Affymetrix-probe sets 208305_at and 216836_s_at, respectively). The areas under the curves (AUC) are 0.90 (for PGR) and 0.91 (for HER2). To obtain thresholds for discrimination (between PGR^+^ vs. PGR^-^ and HER2^+^ vs. HER2^-^), the same concepts have been applied as described for the estrogen receptor, see legend and text for details. Mid panels: Box plots for gene expression after RMA-normalization for PGR and HER2, with boxes classified according to IHC (+/-). For numbers of samples see Table 1. Lower panels: Normalized histograms of expression values (blue bars). x-axis: gene expression after RMA-normalization over all samples. Y-axis: estimated PDF. PDFs obtained ‘classically’ from means and standard deviations: green curves, see the dashed lines for individual distributions of IHC^+^ and IHC^-^, respectively. PDF obtained via maximum likelihood: red curve.

In order to evaluate gene expression we assumed expression values, $x_{i}$, to be approximately normally distributed ($N(\mu^{+},\sigma^{+})$ for $ER_{\text{IHC}}^{+}$ and $N(\mu^{-},\sigma^{-})$ for $ER_{\text{IHC}}^{-}$), see Figure 1, lower panel. Adding both normal distributions with proper weights ($\lambda^{+}+\lambda^{-}=1$) yielded a bimodal distribution, see the stacked bars in Figure 1. Likewise, we obtained bimodal distributions for progesterone (gene PGR) and human epidermal growth factor receptor 2 (HER2, gene ERBB2), see Figure 3.

To obtain thresholds discriminating receptor positive versus negative we evaluated 5 different methods: MaxLike, ParEst, LogReg, Youden and ExMax, for maths see the ‘Methods and models for expression of receptor genes’ in ‘Material and methods’. We decided to finally adopt method ExMax, see section ‘Why to adopt ExMax’ in ‘Material and methods’, and obtained the cut-points given in Table 3.

**Table 3:** Distribution parameters and cut-points for receptors ER, PGR and HER2

Receptor	Method	CP	μ^−^	*μ*^+^	σ^−^	σ^+^	λ^−^	*λ*^+^	π^+^
ER	MaxLike	9.453	6.515	11.624	1.415	0.947	0.483	0.517	n/a
	ParEst	9.099	6.835	11.300	1.870	1.580	0.483	0.517	n/a
	LogReg	9.177	n/a	n/a	n/a	n/a	n/a	n/a	n/a
	Youden	8.832	n/a	n/a	n/a	n/a	n/a	n/a	n/a
	**ExMax**	**9.363**	**6.557**	**11.472**	**1.534**	**1.013**	**n/a**	**n/a**	**0.519**
PGR	MaxLike	4.046	3.587	5.560	0.197	1.483	0.542	0.458	n/a
	ParEst	4.399	3.647	5.336	0.409	1.555	0.542	0.458	n/a
	LogReg	4.145	n/a	n/a	n/a	n/a	n/a	n/a	n/a
	Youden	3.929	n/a	n/a	n/a	n/a	n/a	n/a	n/a
	**ExMax**	**4.164**	**3.619**	**5.722**	**0.241**	**1.486**	**n/a**	**n/a**	**0.454**
HER2	MaxLike	11.680	9.481	12.982	0.870	0.585	0.754	0.246	n/a
	ParEst	11.053	9.396	11.912	0.867	1.484	0.754	0.246	n/a
	LogReg	11.101	n/a	n/a	n/a	n/a	n/a	n/a	n/a
	Youden	10.633	n/a	n/a	n/a	n/a	n/a	n/a	n/a
	**ExMax**	**12.304**	**9.492**	**13.209**	**1.251**	**0.538**	**n/a**	**n/a**	**0.124**

Cut-points (CP) as obtained by 5 different methods (MaxLike, ParEst, LogReg, Youden and ExMax), cf. the colored dots in Figure 2 and Figure 3.

Similar results were obtained for PGR and HER2, see the bottom rows in Figure 3. Computationally, we proceeded along the very same lines as for ER. Note that the majority of patients was HER2-. As a consequence, thresholds shifted towards larger expression values (as compared to ER) and specificity of discrimination resulted fairly large.

Although HER2+ has much lower prevalence then HER2-, the ExMax-estimate performs outstandingly well, as can be seen from Figure 3.

### Agreement between receptor status obtained by IHC versus gene expression cut-points

Using the cut-points (CP) shown in Table 3, we obtained receptor status from gene expression as compared to IHC-estimates, see Table 4.

**Table 4:** Agreement (contingency tables) between receptor status from IHC versus gene expression

	estrogen ER		progesterone PGR		HER2	
	GE–	GE+	GE–	GE+	GE–	GE+
IHC−	943	130	892	197	1573	14
IHC+	112	1084	184	487	305	233
log(DOR) ± 95%	4.25±0.27		2.48±0.23		4.45±0.55	
Cohen's Kappa	0.79		0.54		0.52	
Kruscal's Gamma	0.97		0.85		0.98	
Rate of discordant samples	11%		22%		15%	
Rate of conclusive samples	89%		78%		85%	

Cut-points for gene expression were those derived by the ExMax-algorithm, see Table 3. Four measures of agreement are given: log(DOR) (natural logarithm of the Diagnostic Odds Ratio, DOR), Cohen's Kappa (inter-rater agreement), Kruscal's Gamma (strength of association) and the rate of discordant samples (conjugate to the rate of conclusive samples).

Association between IHC and gene expression was quantified by Kruscal's gamma, Cohen's Kappa (cf. Table 4) and also by the diagnostic odds ratio, DOR, and its 95% confidence intervals [21].

Disagreement between IHC and GE, as quantified by the rate of discordant samples, is highly problematic in the clinical setting, and a clinical decision should not be made on the basis of IHC data only.

PGR showed less agreement than ER due to its lower expression values, entailing a broad distribution of *PGR* expression values for ${\text{PGR}}_{\text{IHC}}^{+}$. Regarding HER2, the precision of determination suffered from an imbalance in the data: Much more patients are HER2 negative than positive. Note that missing IHC-estimates, as considered in the next chapter, were not included here.

In order to improve this situation we enhanced the decision process by furnishing GE with a responsibility function and a critical domain.

### Improved decision quality due to responsibility function and critical domain

**Figure 4:**
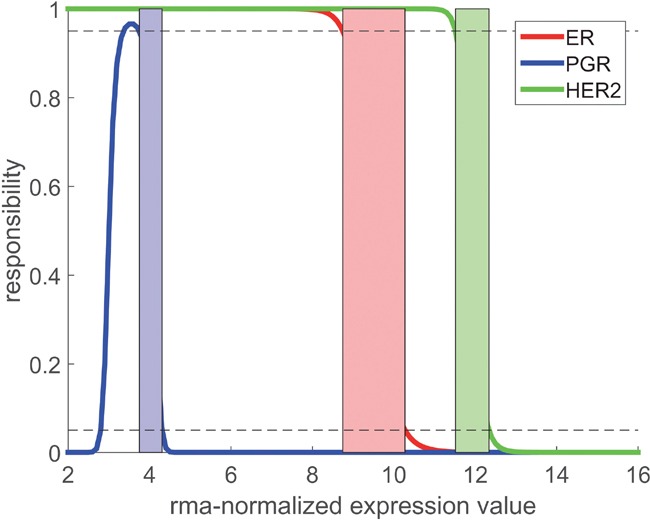
Responsibility functions $r^{-}$ for receptors ER, PGR and HER2 Critical domains, where no decision is possible, are indicated as colored stripes. For PGR, $f^{+}$is extremely wide (very large $\sigma^{+}$) and causes the unexpected shape of $r^{-}$. Borders of critical domains result from the intersections of the respective $r^{-}$ with the 5% and 95% limits (dotted lines).

Instead of using a single cut-point for each receptor we introduced a ‘critical domain’ $\left[X_{\text{lower}},\;X_{\text{upper}}\right]$ for a probability level α=0.05, see Figure 4 and, for mathematical details, the chapter ‘Material and methods’. For expression values $x<X_{\text{lower}}$ we decided for receptor negative, for $x>X_{\text{upper}}$ receptor positive: In both cases we classified the expression value as ‘informative’. As opposed, expression values within the critical domain $X_{\text{lower}}\leq x\leq X_{\text{upper}}$ were classified as ‘undecidable’, and we refrained from a decision. Critical domains thus constructed are given in Table 5 and their practical application is described in section, Expression lookup plot‘ in ‘Material and methods’.

**Table 5:** Critical domains of gene expression for receptor status determination

Receptor	Probeset	X_lower_	X_upper_
ER	205225_at	8.517	10.354
PGR	208305_at	3.664	4.407
HER2	216836_s_at	11.783	13.070

The critical domain for expression values *x* is given by $X_{\text{lower}}\leq x\leq X_{\text{upper}}$, based on the ExMax-algorithm and probe sets listed in Table 2. The section ‘Expression lookup plot’ in ‘Material and methods’ describes how to actually apply the criterion $X_{\text{lower}}\leq x\leq X_{\text{upper}}$

**Figure 5:**
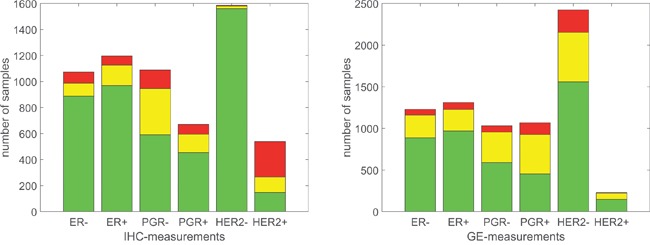
Number of concordant and discordant samples Left panel: Bars according to IHC receptor estimate. Height of bar represents number of samples with gene expression concordant (green), in critical domain (yellow) and discordant (red). Note that samples without IHC estimate are not included. Right panel: Bars according to GE receptor estimate. Height of bar represents number of samples with IHC concordant (green), missing (yellow) and discordant (red).

The information conveyed by gene expression was thus refined, yielding the results for GE 0, shown in Table 6 and the yellow sub-bars in Figure 5.

**Table 6:** Number of samples for which IHC and gene expression yield receptor positive/unknown/negative

	gene expression								
	ER			PGR			HER2		
	GE –	GE 0	GE +	GE –	GE 0	GE +	GE –	GE 0	GE +
IHC –	887	100	**86**	590	357	**142**	1560	20	7
IHC 0	273	81	257	365	282	473	593	88	74
IHC +	**70**	157	969	**75**	143	453	**271**	119	148
Critical GE		338			782			227	
Rate of discordant samples		5%			8%			10%	
Rate of conclusive samples		92%			79%			87%	

IHC 0: no IHC measurement available.

GE 0: expression value within the critical domain, and no decision can be made. Boundaries of critical domains were computed by the ExMax-method.

Bold print: discordant samples.

Note that results shown in this table are obtained step by step via the methods explained in ‘Material and Methods’.

**Table 7:** Distribution parameters of gene expression and their 95% confidence-intervals

Receptor	μ−	μ+	σ−	σ+
ER	6.557 ± 0.047	11.472 ± 0.031	1.534 ± 0.043	1.013 ± 0.027
PGR	3.619 ± 0.007	5.722 ± 0.050	0.241 ± 0.006	1.486 ± 0.031
HER2	9.492 ± 0.047	13.209 ± 0.008	1.251 ± 0.036	0.538 ± 0.008

Parameters have been obtained by the ExMax-method applied to 2880 samples. 95% Confidence intervals have been obtained via expected Fisher information (see ‘Material and methods’, section ‘Obtaining confidence intervals for expression distribution parameters’) and verified by bootstrapping (see chapter 8 in [35]).

A critical domain regarding GE indicates whether or not to trust in GE expression alone. This is especially useful in cases of lacking IHC estimates. For ER in particular, 81 cases revealed as untrustworthy, since missing IHC was accompanied by GE within the critical domain.

Introducing a critical domain is in particular useful in the case of PGR, since GE estimates of PGR offer only limited power of discrimination (leaving as many as 782 samples in column GE 0). But also for ER, 338 samples were revealed untrustworthy (column GE 0).

HER2 estimates revealed a special problem: Many ${\text{HER2}}_{\text{IHC}}^{+}$ samples had low (i.e. contradicting) GE, suggesting they might be false positives from IHC measurement, see the red part of the rightmost bar in left panel of Figure 5.

### Therapy allocation check and consequences

**Figure 6:**
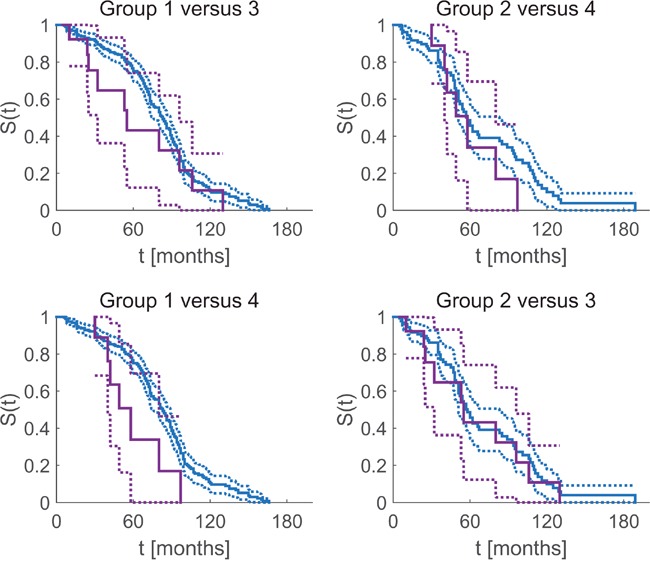
Correct and possibly miss-led assignment of systemic therapy influences survival time free of symptoms Kaplan Meier estimate of overall survival and 95% confidence intervals. Blue curves: patients with IHC status in accordance with gene expression (correctly assigned therapy). Violet: patients with IHC status contradicted by GE and possibly having received suboptimal therapy.

In order to scrutinize if the additional information conveyed by GE might change therapeutic decisions based on IHC only, we compared four groups:

1. $\left(ER_{\text{IHC}}^{+}\wedge ER_{\mathrm{GE}}^{+,0}\right)\vee \left({\mathrm{PGR}}_{\text{IHC}}^{+}\wedge {\mathrm{PGR}}_{\mathrm{GE}}^{+,0}\right)$ (see Figure 6, blue curves, left column, 329 Pat.): Receptor positive patients according to IHC, had received hormone treatment. Since GE confirmed IHC, the choice of systemic therapy deems correct in these cases.

2. $\left(ER_{\text{IHC}}^{-}\wedge {\mathrm{PGR}}_{\text{IHC}}^{-}\right)\wedge \left(ER_{\mathrm{GE}}^{0,-}\wedge {\mathrm{PGR}}_{\mathrm{GE}}^{0,-}\right)$ (see Figure 6, blue curves, right column, 137 Pat.): Receptor negative patients according to IHC, had not received hormone treatment, accordingly. Since GE confirmed IHC, the choice of systemic therapy deems correct in these cases.

3. $\left(ER_{\text{IHC}}^{+}\vee {\mathrm{PGR}}_{\text{IHC}}^{+}\right)\wedge \left(ER_{\mathrm{GE}}^{-}\wedge {\mathrm{PGR}}_{\mathrm{GE}}^{-}\right)$ (see Figure 6, violet, upper left, lower right, 18 Pat.): Patients deemed receptor positive according to IHC, had received hormone treatment, accordingly. Since GE contradicted IHC, the choice of systemic therapy might have been incorrect in two possible ways: Those patients who had received neoadjuvant hormone therapy (in addition to adjuvant chemotherapy) would not have benefitted from it (negligible harm). Those patients receiving only hormone therapy without chemotherapy would have been given an ineffective therapy while at the same time being deprived of the adequate, live-saving therapy.

4. $\left(ER_{\text{IHC}}^{-}\wedge {\mathrm{PGR}}_{\text{IHC}}^{-}\right)\wedge \left(ER_{\mathrm{GE}}^{+}\vee {\mathrm{PGR}}_{\mathrm{GE}}^{+}\right)$ (see Figure 6, violet, upper right, lower left, 9 pat.): Patients deemed receptor negative according to IHC, not having received hormone treatment, accordingly. Since GE contradicted IHC, the choice of systemic therapy might have been suboptimal in these cases.

For each group, we calculated the Kaplan Meier product limit estimator for time free from relapse (see Figure 6) and obtained 95% confidence limits via the Greenwood formula [22].

In group 1 (blue, left column), a patient's positive hormone-receptor status according to IHC was not contradicted by GE. Accordingly, these patients are likely to have been allocated the ‘correct’ i.e. anti-hormone treatment and have actually benefitted therefrom.

Patients in group 2 (blue, right column) had been assessed hormone receptor negative via IHC, and this was confirmed by GE: They are likely to have received optimum systemic therapy.

In contrast, patients in group 3 (violet, upper left and lower right) had been assessed hormone receptor positive via IHC, however, this was contradicted by GE: they might have received a hormone therapy but may not have benefitted from it (since they are likely to lack receptors, according to GE). It might even be the case that adjuvant chemotherapy was spared although being mandatory, relying on just hormone therapy (which did not work in these patients).

Patients in group 4 (violet, upper right and lower left) had been assessed hormone receptor negative via IHC, however, this was contradicted by GE: They might have been deprived of a hormone therapy they could have benefitted from.

The above grouping is weakly formulated by intention, in the sense that IHC estimates (used as basis for therapeutic decision) were questioned if and only if GE actually contradicted, not if GE was just within its critical domain ([GE+, GE0] for receptor positive, [GE-, GE0] for receptor negative). Tightening the definition of error-prone groups (not tolerating ‘0’ anymore), might even aggravate the contrasts, concomitantly reducing the number of patients in each group, however. We decided to stick to the broader (and weaker) definition so as not to withhold possibly decisive warnings to patients with borderline receptor status.

## DISCUSSION

### Achievements due to responsibility function and critical domain

For the majority of patients (89%), the IHC-assessment of estrogen receptors (ER_IHC_) and gene expression (ER_GE_, gene *ESR1*) data yielded compatible results and we may consider such an estimate true. This percentage was similar to the portion of IHC-estimates in the literature suspected to be true [23]. In this work we paved the way to consistently deal with missing as well as contradicting estimates as follows, see Table 6.

In cases of missing IHC-estimates one cannot simply trust GE. Instead, in order to strengthen decision performance, we rather have to introduce a critical region for GE. It effectively flags those cases where GE alone deems untrustworthy – 81, 282 and 88 for ER, PGR and HER2, respectively – and thereby provides a ‘quality filter’.

Available IHC estimates which are contradicted by GE when based on simple cut-points (crisp decision), represent a large number of discordant cases:

(a) For ER, about (11% of patients in our cohort, ER_IHC_ and ER_GE_ disagreed, with about equal numbers (130/112) being found in each of the two possible modes of disagreement see Table 4.

(b) For PGR, discordant cases were similarly balanced (197/184), the rate of discordant samples (22%) being twice that of ER, however.

(c) For HER2 finally, discordant cases were severely imbalanced (14/305), with a rate of 15% of discordant samples, cf. Table 4 and Table 6.

We have to consider, however, that samples with formerly ‘contradicting’ GE (according to a crisp decision based on a simple cut-point for GE) may switch to non-contradicting, i.e. ‘compliant’, if GE lied just within (and not beyond the limits of) its critical domain.

Thus, considering a critical domain for GE, the rate of discordant samples decreased while the rate of conclusive samples increased, as can be seen by comparing Table 4 and Table 6.

### Results for patients on top of those for statistics

If the goal were just to build a cohort of patients for developing a gene-expression signature statistically, one could, of course, retreat to the save side and consider only cases in agreement. However, even this might bias the results and call for an improvement of receptor estimate quality.

However, if the goal are decisions upon therapeutic interventions, these have to be made for all patients and in utmost quality - even in cases of disagreement between IHC and gene expression. We therefore tried to reduce the risk of wrong decisions by improving the cut-point-method by the additional concept of a ‘critical domain’.

### Improving receptor security for a newly diagnosed patient

For a newly diagnosed patient, we proposed a procedure to improve receptor security, see section ‘Expression lookup plot’ in ‘Material and methods’. The ‘critical domain’ denotes non-decidable cases with responsibilities between 0.05 and 0.95.

Note that such a direct procedure is only possible due to the nature of RMA-normalization of expression data, see section ‘Receptor status obtained from gene expression’ in ‘Results’.

This straight forward procedure for the newly diagnosed patient is easily applicable (and renders any re-normalization on the fly, etc., unnecessary).

Note however, that the ‘expression look-up-plot’ may critically depend on expression chip processing, despite RMA-normalization. We hence recommend building the ‘expression look-up-plot’ on the basis of gene expression measurements performed in the very same environment as data from newly diagnosed patients are analysed.

As a consequence and achievement, treatment quality of newly diagnosed patients will benefit, thus conveying a significant step towards precision medicine.

### Balancing the risks of erroneous receptor status

We have shown that additional information from GE-data including a critical domain may improve receptor security in a considerable portion of cases, see Table 6.

a) ${\text{ER}}_{\text{IHC}}^{-}$

Out of 1073 patients classified as ${\text{ER}}_{\text{IHC}}^{-}$ only 92% were not contradicted by gene expression. Without our proposed re-assessment, the remaining 8% may receive chemotherapy although hormone therapy might suffice. Thus, applying our proposed method, unnecessarily severe side-effects could possibly be saved in approximately 8% of that patient cohort.

b) ${\text{ER}}_{\text{IHC}}^{+}$

Out of 1196 patients classified as ${\text{ER}}_{\text{IHC}}^{+}$ only 94% were not contradicted by gene expression. Without our proposed re-assessment, the remaining 6% may erroneously receive non-effective endocrine therapy while chemotherapy is withheld, which may be lethal. Thus, identifying these patients, may save lives in approximately 6% of that patient cohort.

c) ${\text{PGR}}_{\text{IHC}}^{-}$

Out of 1089 patients classified ${\text{PGR}}_{\text{IHC}}^{-}$ only 87% were not contradicted by gene expression. Without our proposed re-assessment, the remaining 13% would possibly receive under-treatment (if also ${\text{ER}}_{\text{IHC}}^{-}$), since endocrine therapy would be falsely withheld from this population.

d) ${\text{PGR}}_{\text{IHC}}^{+}$

Out of 671 patients classified ${\text{PGR}}_{\text{IHC}}^{+}$ only 89% were not contradicted by gene expression. Without our proposed re-assessment, the remaining 11% would be falsely misclassified into endocrine sensitive and would thus be exposed to the side effects of endocrine therapy without a therapeutic benefit.

e) ${\text{HER2}}_{\text{IHC}}^{-}$

Out of 1587 patients classified ${\text{HER2}}_{\text{IHC}}^{-}$, 99.6% were not contradicted by gene expression. Without our proposed re-assessment, the remaining 0.4% would be exposed to Herceptin-associated side effects without deriving a benefit from this expensive and side-effect-related therapy.

f) ${\text{HER2}}_{\text{IHC}}^{+}$

Out of 538 patients classified ${\text{HER2}}_{\text{IHC}}^{+}$ only 50% were not contradicted by gene expression. Without our proposed re-assessment, the remaining 50% would not be able to receive HER2-targeted treatment such as Herceptin, and would be under-treated.

Balancing the consequences of above listed decisions in therapy allocation we adhere to the general consensus that withholding endocrine therapy when it should be delivered is more harmful than applying endocrine therapy when not needed. Hence,

· the therapy allocation error in c) is more serious than it is in d).

· the therapy allocation error in f) is more serious than it is in e).

· the most serious allocation error is listed in b), concerning patients falsely diagnosed as receptor positive.

### Characteristics of selected procedures

This section critically enumerates assumptions underlying the results presented here.

### Dependence of procedures on IHC - measurements

Expectation maximization (ExMax) does not draw on IHC-measurements at all, i.e. all 5 parameters of the two normal distributions ($\pi^{+},\mu^{+},\sigma^{+},\mu^{-},\sigma^{-}$)^1^ are estimated simultaneously from expression data, see Table 3. On the contrary, for methods Youden, ParEst, LogReg and MaxLike, $\lambda^{+}$ has to be computed from IHC-values in advance (i.e. these methods depend on IHC-measurements).

^1^$\lambda^{+}$ and $\lambda^{-}$ are normalized to unity, hence only $\lambda^{+}$ is free to be adjusted.

### Dichotomous IHC-estimates

IHC assays initially produce raw values which are normally dichotomized into $ER_{\text{IHC}}^{+}$ and $ER_{\text{IHC}}^{-}$ according to some thresholds. Using more than one threshold yields results such as ‘low’, ‘moderate’, ‘high’, etc.

A straight forward improvement of the current methodology could draw on raw values of IHC-assays and file them into the analysis as real values similar to gene expression values. Such an approach could even outperform the one presented here and may be the scope of future investigations.

A drawback of this proposal is, however, that in most cases we only have dichotomized IHC-estimates at hand. No large datasets are available on the net to pursue the issue if gradual IHC estimates could improve precision.

### Goals achieved and clinical impact

Receptor status estimated via IHC is a key ingredient of precision medicine for breast cancer patients. However, significant doubts have been raised regarding reliability, when comparing disparate receptor estimates of different readers evaluating the same data [5].

On the other hand, receptor estimates from gene expression, which are usually not used in clinical settings, have been given even more credit than IHC estimates [24].

Taking GE in account in addition to IHC clearly boosts credibility of concordant cases but, at first glance, reduced the number of decidable cases, since contradicting cases also emerged. However, in a second step, concordance could be re-improved by considering a critical domain of GE, thereby again reducing the number of contradictory cases.

In this work we scrutinized possible mathematical ways to enrich receptor information from IHC by gene expression data and thus put decisions on therapies on more solid grounds. To these ends we developed a procedure to incorporate both, IHC estimates and gene expression data.

This work used Affymetrix data together with clinical parameters downloaded from GEO in search for an improved receptor status determination. Translated into clinical reality, the expression status of those few genes involved will most probably be determined via RT-qPCR. Gene expression for single, newly diagnosed patients can be made interpretable and meaningful by our proposed ‘expression-lookup-table’ and lends itself as a valuable tool for improved receptor status assessment.

## MATERIALS AND METHODS

### Data for gene expression and receptor status

Scanning the gene expression omnibus, GEO [25–27], for studies on breast cancer, using Affymetrix U133A+2.0 arrays and also containing receptor data resulted in 28 studies, two of which are redundant. The 26 non-redundant studies comprise a total number of *N*_sample_ = 2880 individual breast cancer samples, after excluding samples completely lacking clinical information, control samples as well as duplicate samples.

Affymetrix-probe sets for receptors are given in Table 2.

### Normalization

RMA-normalization was performed on all 54675 probe sets on the Affymetrix U133A+2.0 expression chip using the rma-function from the Bioconductor's affy- package [28–30].

For the analyses we restricted ourselves to the subset of the 22215 probe sets U133A+2.0 has in common with U133A, in order to be downwards compatible.

### Methods and models for expression of receptor genes

To exploit information from the expression of receptor genes we considered five methods: MaxLike, ParEst, LogReg, Youden and ExMax.

#### Method 1: Youden point

The conventional, most straight forward approach is to compute the Youden point [31] along the ROC-curve by maximizing

J=sensitivity+specificity−1 (1)

see the label ‘Youden’ in Figure 1, Figure 2 and Figure 3, lower panels. The expanded formula reads:

$$J=\frac{\text{true positives}}{\text{true positives}+\text{false positives}}+\frac{\text{true negatives}}{\text{true negatives}+\text{false positives}}$$ (2)

#### Method 2: logistic regression (LogReg)

Another simple approach is logistic regression [32] of receptor status versus expression values, $x_{i}$. The cut-point is set at equal probabilities (0.5, 0.5) for receptor positive and negative, respectively, see the label ‘LogReg’ in Figure 1, Figure 2 and Figure 3, lower panels. Parameters of distributions ($N(\mu^{+},\sigma^{+})$ and $N(\mu^{-},\sigma^{-})$) are not considered.

#### Method 3: parameter estimation from bimodal distribution (ParEst)

Normal distributions are evaluated classically by computing mean and standard deviation $\left(\mu^{+},\sigma^{+}\right)$ from $N^{+}$ receptor positive patients and $\left(\mu^{-},\sigma^{-}\right)$ from $N^{-}$ receptor negative patients, see the dashed green curves in Figure 1, Figure 2 and Figure 3. Weights ($\lambda^{+}=N^{+}/\left(N^{+}+N^{-}\right)$, $\lambda^{-}=N^{-}/\left(N^{+}+N^{-}\right)$) are preset from relative abundances of IHC-measurements (not fitted). This yields the overall probability-density functions (PDF, solid green line).

$$f\left(x\right)=\lambda^{+}f^{+}\left(\mu^{+},\sigma^{+}|x\right)+\lambda^{-}f^{-}\left(\mu^{-},\sigma^{-}|x\right)$$ (3)

The threshold is set at equal Bayesian probabilities Pr(+|x)=Pr(−|x)=0.5 [33], where the Bayesian probability is given by:

$$\mathrm{Pr}\left(A|x\right)=\frac{f^{\left(A\right)}\left(x\right)\cdot \lambda^{\left(A\right)}}{\sum_{A}f^{\left(A\right)}\left(x\right)\cdot \lambda^{\left(A\right)}}$$ (4)

Here $f^{\left(A\right)}$ represents the probability density for receptor-status A∈{−,+} and *x* is the measured expression value of the respective gene.

Equating Pr(+|x)=Pr(−|x) yields the following solution for the cut-point *x*:

$$\begin{array}{cc} a=\frac{1}{{\left(\sigma^{-}\right)}^{2}}-\frac{1}{{\left(\sigma^{+}\right)}^{2}} & b=-2\left(\frac{\mu^{-}}{{\left(\sigma^{-}\right)}^{2}}-\frac{\mu^{+}}{{\left(\sigma^{+}\right)}^{2}}\right)\\ c={\left(\frac{\mu^{-}}{\sigma^{-}}\right)}^{2}-\frac{{\left(\mu^{+}\right)}^{2}}{{\left(\sigma^{+}\right)}^{2}}+\log\left(\frac{\sigma^{-}}{\sigma^{+}}\frac{\lambda^{+}}{\lambda^{-}}\right) & x=\frac{-b+\sqrt{b^{2}-4\mathrm{ac}}}{2a} \end{array}$$ (5)

#### Method 4: maximum likelihood estimator of bimodal distribution (MaxLike)

Weights of both normal distributions are preset (not fitted), according to relative abundance $N^{+}/N^{-}$, as in ‘ParEst’. Distribution parameters $\left(\mu^{+},\sigma^{+}\right)$ and $\left(\mu^{-},\sigma^{-}\right)$ are then computed as the maximum likelihood estimator [34] via a 4 parameter fit (gradient descent method), directly from the bimodal gene-expression histogram. This fit has to act upon a very flat target function, and therefore we implemented the Newton-gradient algorithm to draw on analytical derivatives.

$$L\left(\mu^{+},\sigma^{+},\mu^{-},\sigma^{-}|\vec{x}\right)=\prod_{j=1}^{N_{\text{sample}}}\left(\lambda^{+}f^{+}\left(\mu^{+},\sigma^{+}|x_{j}\right)+\lambda^{-}f^{-}\left(\mu^{-},\sigma^{-}|x_{j}\right)\right)\to \max$$ (6)

$$\mathrm{Pr}\left(A|x\right)=\frac{f^{\left(A\right)}\left(x\right)\cdot \lambda^{\left(A\right)}}{\sum_{A}f^{\left(A\right)}\left(x\right)\cdot \lambda^{\left(A\right)}}$$ (7)

Computing the cut-point is achieved again via eq. (4) and eq. (5).

#### Method 5: expectation maximization (ExMax)

The ExMax-algorithm is a general and very potent mathematical concept, see p. 275 in [35], which can fruitfully be broken down and applied to receptor estimation, as follows.

ExMax not only estimates the parameters of both normal distributions, $\left(\mu^{+},\sigma^{+}\right)$ and $\left(\mu^{-},\sigma^{-}\right)$, but also their relative weight as an additional parameter, $\pi^{+}$, yielding $\left(\pi^{-}=1-\pi^{+}\right)$ due to normalization. Hence, ExMax is able to work without any IHC-estimates.

In short the process is as follows: We start from an initial guess for parameters of the two normal distributions ($\left({\hat{\mu }}_{1},{\hat{\sigma }}_{1}\right)$, $\left({\hat{\mu }}_{2},{\hat{\sigma }}_{2}\right)$ and ${\hat{\pi }}^{+}=N^{+}/\left(N^{+}+N^{-}\right)$. It is only this initial guess – not the data used later for the actual estimation – which may be obtained from IHC-data.

Instead of maximizing the likelihood straight away (as in method 4), it is expanded by additional $N_{\text{sample}}$ latent parameters

$${\hat{\gamma }}_{i},\;i=1....N_{\text{sample}}$$ (8)

${\hat{\gamma }}_{i}$ represents a guess (probability) for sample *i* to be receptor-positive.

a) These guesses are computed (via a special formula) from the guesses for the ‘real’ parameters of the two normal distributions $\left({\hat{\mu }}_{1},{\hat{\sigma }}_{1}\right)$, $\left({\hat{\mu }}_{2},{\hat{\sigma }}_{2}\right)$ and ${\hat{\pi }}^{+}=N^{+}/\left(N^{+}+N^{-}\right)$.

b) From this expanded maximum likelihood function, new estimates for the ‘real’ parameters are obtained via maximization.

Steps a) and b) are repeated in a loop until convergence. Probability density functions based on these fitted parameters are shown as blue lines in Figure 1, Figure 2 and Figure 3.

Cut-points are obtained via eq. (4) and eq. (5), but λ's are replaced by calculated π’s.

Note that IHC-results need to be known in advance and individually for each patient to apply methods Youden, LogReg and ParEst (i.e. supervised methods). As opposed, MaxLike draws on the relative abundances $\lambda^{+}=N^{+}/\left(N^{+}+N^{-}\right)$ only (semi-supervised method). ExMax does not need IHC data at all (unsupervised method), and hence lends itself for analyzing cohorts totally lacking IHC information.

### Why to adopt ExMax

Screening the five methods resulted in the following valuation:

· LogReg draws on IHC estimates which may partly be wrong, possibly blurring the decision.

· Youden yields only a cut-point without a confidence interval.

· MaxLike and ExMax yield almost identical results. However, according to our tests, MaxLike seems to perform well only if the abundances of false positive and false negative estimates are about equal, which is incidentally the case in our data. As opposed, ExMax works unaffected by relative abundances. Hence, we preferred ExMax over MaxLike.

· ParEst yields larger confidence intervals than ExMax, see section ‘Obtaining confidence intervals for expression distribution parameters’.

· LogReg and Youden do not draw on normal distributions. Finally, each method yields a specific cut-point for classifying the expression value, $x_{i}$, see the marks on the ROC-curve (Figure 2), corresponding to the vertical lines in Figure 1 and Figure 3, lower panels.

All proposed methods were found to yield compatible cut-points, see Table 3, and ExMax was finally selected. It yields a PDF ($f^{-}$) for receptor negative and a PDF ($f^{+}$) for receptor positive.

### Obtaining confidence intervals for expression distribution parameters

For the methods ParEst, MaxLike and ExMax, outlined to model receptor gene expression, the accuracy of parameters can be estimated by several methods, such as bootstrap, Leave-One-Out (LOO), cross-validation (CV, via training and validation set) or else by Fisher's information criterion, FIC, [35]. We will stick to FIC, and it works as follows:

We start from the likelihood function, plug in all parameters (means and standard deviations) as a ‘vector of parameters’, $\vec{\theta }$, and get:

$$L\left(\mu^{+},\sigma^{+},\mu^{-},\sigma^{-}|\vec{x}\right)=L\left(\vec{\theta }|\vec{x}\right)$$ (9)

The so-called Fisher Information matrix is obtained as second partial derivative:

$$I(\vec{\theta })=-\sum_{i=1}^{N_{\text{sample}}}\frac{\partial^{2}\text{log}L\left(\vec{\theta }|{\vec{x}}_{i}\right)}{\partial \vec{\theta }\;\partial {\vec{\theta }}^{T}}$$ (10)

Next, we would like to evaluate the expectation value of $I(\vec{\theta })$ by integrating over all measured values:

$$i(\vec{\theta })=\int I\left(\vec{\theta }|{\vec{x}}_{i}\right)\cdot f\left(\vec{\theta }|{\vec{x}}_{i}\right)d\vec{x}$$ (11)

For a bimodal normal distribution this is not feasible. As an approximation, we instead evaluate $I(\vec{\theta })$ for the maximum likelihood estimate of the parameters, $\hat{\vec{\theta }}$, as arguments: $I\left(\hat{\vec{\theta }}|\vec{x}\right)$. It can be shown mathematically [35] that this converges towards the expectation value.

From statistics one knows that the parameter estimates are normally distributed

$$\hat{\vec{\theta }}\approx N\left(\hat{\vec{\theta }},\;i{\left(\hat{\vec{\theta }}\right)}^{-1}\right)$$ (12)

We take the main diagonal of this matrix and therefrom construct 95% confidence limits through multiplication by 1.96

$${\hat{\vec{\theta }}}_{j}\pm 1.96\cdot \sqrt{{\left(i{\left(\hat{\vec{\theta }}\right)}^{-1}\right)}_{\mathrm{jj}}}$$ (13)

For results see Tˆable 7.ˆ

### Responsibility functions define lower and upper bounds for gene expression

**Figure 7:**
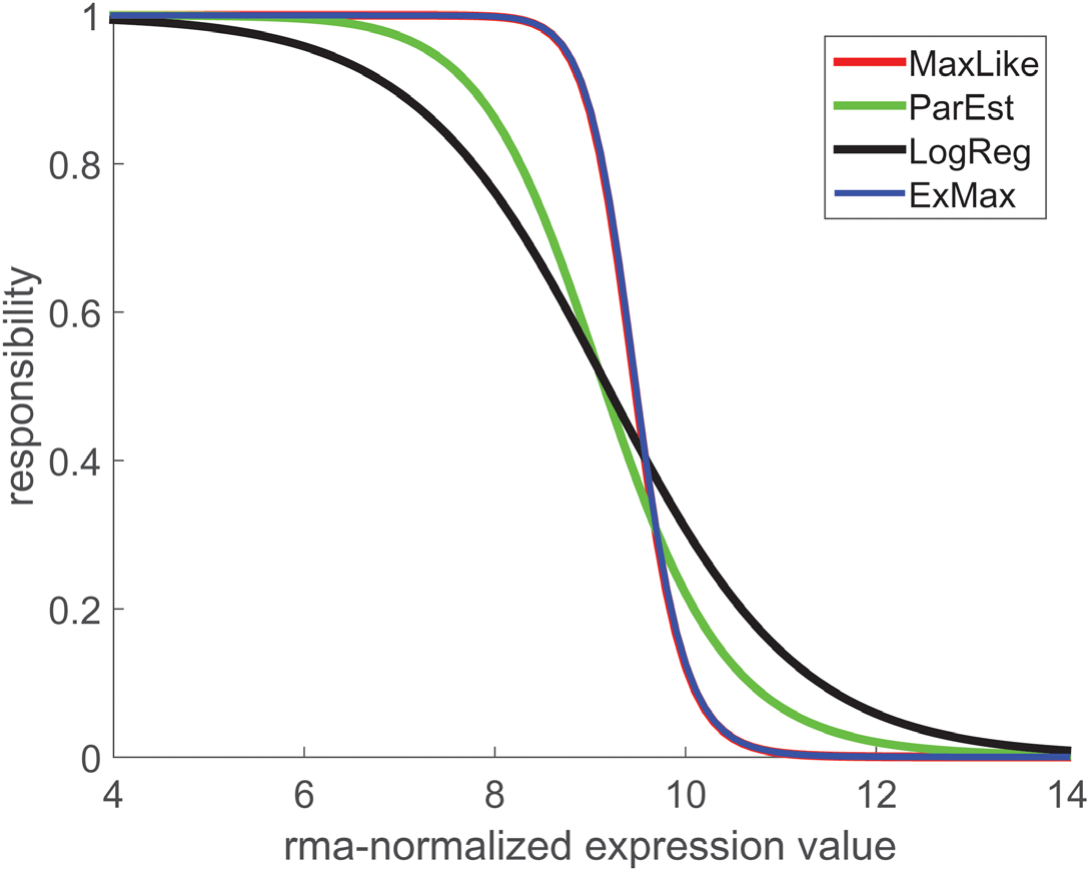
Responsibility functions for estrogen receptor-negative Each of the four computational concepts (see legend) yields a separate responsibility function, $r^{-}$, showing the probability that the sample from a given patient is estrogen receptor-negative, based on the gene expression value. A responsibility = 0.5 determines the cut-point in terms of gene expression. Note that the methods MaxLike and ExMax yield coinciding curves (blue and red, appearing as violet). It becomes quite obvious: the steeper the responsibility function declines, the more accurate the limits $\left[X_{\text{lower}},\;X_{\text{upper}}\right]$ can be determined. In any case $r^{{}_{-}}(x)$ is monotonously declining from 1 to 0.

Each of the four estimation methods (LogReg, ParEst, MaxLike, ExMax) lends itself to construct a ‘responsibility function’, $r^{-}(x)$, for the respective sample being receptor-negative (likewise, $r^{+}(x)$ can be constructed for a sample being receptor positive). Each method yields a separate responsibility function, see Figure 7.

For the methods ParEst, MaxLike and in particular for ExMax, which we finally adopted, responsibility functions are constructed as follows:

Given the two PDFs $f^{\;-}$ and $f^{\;+}$ (see Figure 1 and Figure 3), two ‘responsibility functions’ are constructed

$$r^{-}=\frac{f^{-}}{f^{-}+f^{+}}\text{and}r^{+}=\frac{f^{+}}{f^{-}+f^{+}}$$ (14)

and may be directly expressed in terms of the distribution parameters:

$$\begin{array}{c} r^{-}(x)=\frac{\left(1-\pi^{+}\right)\cdot f\left(x|\mu^{-},\sigma^{-}\right)}{\left(1-\pi^{+}\right)\cdot f\left(x|\mu^{-},\sigma^{-}\right)+\pi^{+}\cdot f\left(x|\mu^{+},\sigma^{+}\right)}\\ r^{+}(x)=\frac{\pi^{+}\cdot f\left(x|\mu_{+},\sigma_{+}\right)}{\left(1-\pi^{+}\right)\cdot f\left(x|\mu^{-},\sigma^{-}\right)+\pi^{+}\cdot f\left(x|\mu^{+},\sigma^{+}\right)} \end{array}$$ (15)

The Bayesian concept enters via interpreting $\pi^{+}$ as an *a priori* probability of positive (and $1-\pi^{+}$ of negative) receptor status for a given patient. In other words, responsibility functions indicate if the positive or the negative mode ($r^{+}\left(x\right)$ or $r^{-}\left(x\right)$) is better suited to explain a measured expression value, *x*. Boundaries of the critical region $\left[X_{\text{lower}},\;X_{\text{upper}}\right]$ are obtained by putting

$$r^{-}\left(X_{\text{lower}}\right)=r^{+}\left(X_{\text{upper}}\right)=1-\alpha$$ (16)

The ‘critical domain’ denotes non-decidable cases with responsibilities between 0.05 and 0.95.

### Expression lookup plot

**Figure 8:**
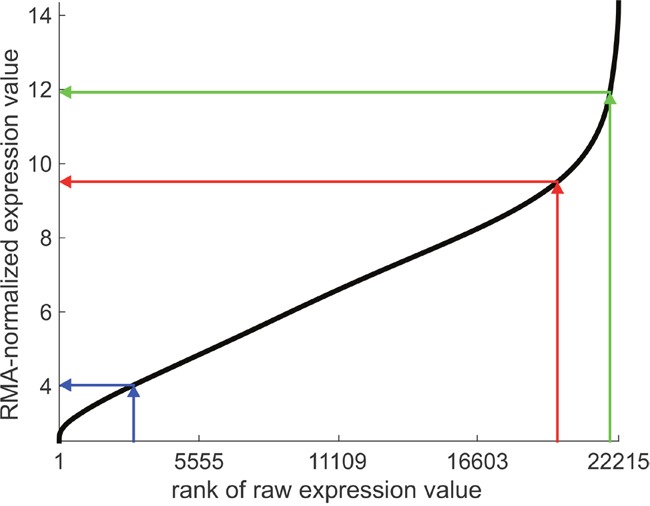
Expression look-up-plot: expression versus rank for the estrogen receptor Probes (*N*_probes =_ 22.215) are ranked according to expression on the x-axis. Arrows indicate the proposed procedure for the newly diagnosed patient: The rank of a receptor gene is embedded within the ranks of all other genes for the very same sample, projected against the curve and the normalized expression value read off the y-axis. This yields a processed value ready for decision making. Values are given for Affymetrix U133A+2.0. Projections for three different ranks are shown: 18.515 → 8.05 (red), 4.319 → 4.75 (blue), 21.917 → 11.19 (green).

As a prearrangement take a gene expression database of a similar patient cohort and some expression platform, e.g. like the one we have used in this work, as a basis. Perform RMA-normalization as described and generate an ‘expression look-up-plot’ similar to Figure 8. It serves as a reference in the following procedure for the newly diagnosed patient:

a) Take the raw expression values of all probe sets, order them by size and obtain ranks 1 – 22.215 (This number depends on the platform used, here a subset of Affymetrix U133A+2.0 as mentioned in section ‘Normalization’).

b) Locate the rank *r* of the receptor probe set (e.g. *ESR1*) among the others e.g. rank 18.515 (within that sample), see Figure 8.

c) Within the ‘expression look-up-plot’ locate the rank of the receptor gene on the rank-axis (x-axis), project it onto the curve and read off the value from the expression axis (y-axis). In math terms, one computes the *x* = *r* / *N*_probes_ - quantile.

d) This value, *x*, comes already in correct dimensions and may directly be compared with the limits of the ‘critical domain’ $\left[X_{\text{lower}},\;X_{\text{upper}}\right]$.

Thus the expression lookup plot yields information directly relevant for improving decision quality based on receptor status.

## Abbreviations

IHC: Immunohistochemistry
GE: Gene Expression
ER: Estrogen Receptors
PGR: Progesterone
HER2: Human epidermal growth factor 2
U133A+2.0: Affymetrix Human Genome U133A 2.0 Gene Expression Array
U133A: Affymetrix Human Genome U133A Gene Expression Array
GEO: Gene Expression Omnibus
ESR1: HUGO gene name for ER
RMA- normalization: Robust Multi-array Average normalization
ROC curve: Receiver-Operating-Characteristic curve
CP: Cut-Point
PDFs: Probability Density Functions
DOR: Diagnostic Odds Ratio
ER_IHC_: Estrogen Receptor, assessed by IHC
ER_GE_: Estrogen Receptor, assessed by Gene Expression
ExMax: Expectation Maximization
LogReg: Logistic Regression
ParEst: Parameter Estimation of bimodal distribution
MaxLike: Maximum Likelihood estimator of bimodal distribution
RT-qPCR: Real-Time quantitative Polymerase Chain Reaction
LOO: Leave-One-Out
CV: Cross-Validation
AUC: Area Under the Curve
HUGO: HUman Genome Organisation
ERBB2: HUGO gene name for HER2
